# Codeine-induced sperm DNA damage is mediated predominantly by oxidative stress rather than apoptosis

**DOI:** 10.1080/13510002.2020.1752003

**Published:** 2020-04-14

**Authors:** Ayodeji Folorunso Ajayi, Roland Eghoghosoa Akhigbe

**Affiliations:** Department of Physiology, College of Medicine, Ladoke Akintola University of Technology, Ogbomoso, Nigeria

**Keywords:** Codeine, opioids, sperm DNA fragmentation, oxidative stress, caspase, 8OHdG, testosterone, infertility, fertility

## Abstract

**Background:** Opioids have been implicated to induce infertility. Although codeine remains the most used opioid for recreational purpose, no study has documented its effect on sperm quality. Elucidating the effect of codeine on sperm cells and the associated mechanisms may provide an insight into preventing drug-induced sperm damage. Twenty-one New Zealand white rabbits were randomized into three groups; control and codeine-treated. The codeine-treated groups received either 4 or 10mg/kg b.w of codeine for six weeks.

**Results:** Codeine treatment led to significant decrease in sperm count, motility, viability, normal morphology, and sperm membrane integrity. This was associated with significant rise in sperm DNA fragmentation, oxidative damage, and caspase 3 activity. The percentage of sperm DNA fragmentation correlates positively with 8-hydroxy-2'-deoxyguanosine, a biomarker of oxidative DNA damage, and caspase 3 activity, a biomarker of apoptosis. The observed correlation was stronger between sperm DNA fragmentation and oxidative DNA damage than sperm DNA fragmentation and caspase 3 activity.

**Conclusion:** This study revealed that chronic codeine exposure causes sperm DNA fragmentation and poor sperm quality primarily via oxidative stress rather than activation of caspase 3-dependent apoptosis. Findings of the present study may explain drug-induced male factor infertility, particularly, those associated with opioid use.

## Background

Available data suggest an increasing incidence of infertility from male-factor. Over the past few years, cases of infertility arising from male factor have drastically increased from 20 to 30% to 50% [1–3]. This is commonly due to reduced libido, premature ejaculation or poor sperm quality. In some cases, the cause seems unclear and thus diagnosed as idiopathic infertility [4]. Several idiopathic cases have been linked with sperm DNA damage [5]. This increases the prevalence of infertility secondary to poor sperm quality. Although spermatogenesis is under the influence of the hypothalamic-pituitary-testicular axis [6], exogenous factors like infections [5], exposure to heavy metals [7–11], cigarette smoking [12–14], irradiation [15–18], chemicals/herbs and drugs [1,19,20] impair spermatogenesis and/or predispose the sperm cells to damage.

Exposure to certain drugs, especially drugs of abuse, plays a vital role in the aetiopathogenesis of sperm damage and male infertility. It is not unlikely that recreational drugs account for the sperm DNA damage seen in idiopathic infertility. This has led to the consideration of these drugs when assessing the aetiology of infertility [21]. *Cannabis sativa* (marijuana), the most widely abused recreational drug, has been reported to have a spermato-toxic effect. Alagbonsi and his colleagues [22,23] observed that *Cannabis sativa* significantly reduced sperm motility and increased the number of abnormal sperm via oxidative-stress. These authors also demonstrated the effect of Δ^9^-tetrahydrocannabinol, an active compound in *Cannabis sativa,* on sperm kinetics. Δ^9^-tetrahydrocannabinol was shown to impair sperm motility, average path velocity (VAP), curvilinear velocity (VCL), straight-line velocity (VSL), the amplitude of lateral head displacement (ALH), and beat cross frequency (BCF) [24].

Similarly, tramadol, a common opioid of abuse, has been shown to significantly reduce sperm count, sperm viability, and normal morphology [25]. Azari *et al.* [26] observed that intraperitoneal administration of tramadol at 10 and 20 mg/kg body weight decreased sperm concentration, motility and vitality in rats. Esua *et al.* [27] demonstrated that at 50 and 100 mg/kg body weight, oral administration of tramadol also led to a significant decline in sperm quality.

Although codeine is the most commonly abused opioid [28,29], and often referred to as the gateway to substance abuse [29], the effect of codeine on sperm quality has not been reported. In the light of this, it is essential to ascertain whether codeine use predisposes to decline in sperm quality, and the possible role of oxidative stress and intrinsic apoptotic cascade.

## Methods

### Drugs and chemicals

Codeine was kindly donated by the National Drug Law Enforcement Agency (NDLEA), Nigeria, for research purpose only. Assays were done using standard ELISA kits. All other reagents used, unless otherwise indicated, were of analytical grade and obtained from Sigma Chemical Co, USA.

### Laboratory animal

Twenty-one (21) adult, apparently healthy male New Zealand White rabbits (*Oryctolagus cuniculus*), aged 12 ± 2 weeks and weighing 900–1000 g were obtained from the Animal House of the Department of Animal Husbandry, Osun State University, Ejigbo, Osun State. They were given access to chow and water *ad libitum* and exposed to 12/12-hr light/dark cycle. The animals were acclimatized for a week. The animals were accord humane care according to the standard outlined in the Guide for the Care and Use of Laboratory Animals’ put together by the National Academy of Science (NAS) and published by the National Institute of Health. The experiment was carried out under the US NAS guidelines.

### Experimental design and drug administration

The rabbits were randomly allotted into 3 groups (*n* = 7): control (1 ml of normal saline), low-dose codeine (4 mg/kg b.w of codeine), and high-dose codeine (10 mg/kg b.w of codeine). Codeine was orally administered using an oro-pharyngeal cannula, once daily for six weeks (between 7:00 am, and 8:00 am), while the control received normal saline as the vehicle. The low dose (4 mg/kg) was based on the Human Equivalent Dose of 1.2 mg/kg daily in three divided doses using the FDA Draft Guidelines, while the high dose (10 mg/kg) derived from the dose–response curves to obtain a submaximal peak dose from our pilot study. This is as reported in our previous study [30].

Twenty four hours after the last treatment, following an overnight fast, the animals were sacrificed after intraperitoneal administration of 5% ketamine (35 mg/kg) and 2% xylazine (5 mg/kg) [30]. The blood samples were obtained via cardiac puncture and centrifuged at 3000 rpm for 10 min, and the serum separated for hormonal assay. The left testis and epididymis were harvested for semen analysis. The right testis of each rabbit was also harvested; the surrounding structures including the adipose tissues were trimmed off, and homogenized in the phosphate buffer and centrifuged at 10,000 rpm for 15 min at 4°C to obtain the supernatant for biochemical assay.

### Assessment of sperm parameters

Sperm parameters were determined by standard methods following the guidelines of the World Health Organization (WHO) [31] with some modifications using a light microscope. The left caudal epididymis was placed in a clean petri dish containing 2 ml of normal saline solution. An incision of about 1 mm was made on the caudal epididymis to liberate its spermatozoa into the saline solution. Motility was determined using four categories of movement; fast progressive, slowly progressive, non-progressive and immotile, concentration was determined in duplicate using Improved Neubauer Haemocytometer (LABART, Germany), and morphology was determined using eosin-nigrosin staining technique.

Briefly, for determination of sperm motility, 10 µl of epididymal sperm suspension was placed on a clean pre-warned slide, mixed with 2 drops of 2.9% sodium citrate and covered with a coverslip [32]. This was examined under the microscope using the × 10 objective.

Sperm count was determined by placing the right caudal epididymis from each rabbit was immersed in 5 ml of formo-saline. An incision of about 1 mm was made on the caudal epididymis to liberate its spermatozoa into the solution. 10 µl of the suspension was transferred to each chambers of the Improved Neubauer Haemocytometer making use of a Pasteur pipette by touching the edge of the coverslip and allowing each chamber to be filled by capillary action. The sperm count was examined by evaluating different fields under the light microscope at a magnification of × 100.

Sperm viability and morphology studies were carried out using eosin-nigrosin stain. Briefly, 10 µl of the epididymal sperm suspension was mixed with 20 µl of eosin and 30 µl of nigrosin dye. A thin smear was made, air-dried, and cover-slipped. The slides were observed under a light microscope using the  × 40 objective. The viable sperm cells were unstained (they did not pick up the stain) while the dead sperm cells were stained (they picked up the stain indicating damaged membrane). A hundred cells per slide were counted to obtain the percentage live/dead ratio. Sperm abnormalities were determined in 400 sperm cells using established methods [33]. A drop each of Wells and Awa stain and epididymal sperm suspension were placed on a warm slide and mixed. A smear was then made, stained, air-dried, and viewed under light microscope. The defects were observed and classified as described by Bloom [34] and Parkinson [35].

### Assessment of sperm plasma membrane integrity using the hypo-osmotic swelling test (HOST)

HOST was carried out following established methods [36,37]. The hypo-osmotic solution was prepared by adding 0.735 g of sodium citrate dehydrate and 1.351 g of fructose in 100 ml of distilled water. 0.1 ml of well-mixed epididymal sperm suspension was added to 1 ml of the hypo-osmotic solution. The sperm suspension was gently mixed by drawing the sample in and out of the pipette and then incubated at 37°C for 30–60 min. After incubation, a drop of the mixture was placed on a glass slide and covered with a clean coverslip. The slide was viewed under  × 40 lens to observe sperm tail swelling. Sperm with swollen tails (normal spermatozoa) were regarded as HOS Positive/reactive, while those without swollen tails (abnormal spermatozoa) were regarded as HOS Negative. One hundred sperm cells were counted from which the percentage of HOS positive cells were determined.

### Extraction of the sperm cell mitochondrial fraction

Epididymal sperm was prepared as previously reported [38]. The epididymal sperm suspension was centrifuged at 600 rpm for 5 min at 4°C. The supernatant (mitochondrial fraction) was frozen at −20°C and stored for further analysis. The pellet (spermatozoa) was washed and then centrifuged two more times with PBS. The obtained aliquots from the pellets were frozen at −20°C, while some aliquots were re-suspended in 1% SDS before being frozen.

### Assessment of lipid peroxidation and antioxidant in sperm cells

Malondialdehyde (MDA), an index of lipid peroxidation, was determined by measuring the thiobarbituric acid reactive substances (TBARS) produced during lipid peroxidation as previously documented [39]. 50 µL of the mitochondrial fraction was deproteinized with 375 µL of Trichloroacetic acid (TCA) and centrifuged at 3000 rpm for 10minutes. 1 mL of 0.75% TBA was added to 0.1 mL of the solution and boiled in a water bath for 20minutes at 100°C and cooled with ice water. The absorbance of the sample/standard was read at 532 nm with a spectrophotometer against the blank. The concentration of TBARS generated was extrapolated from the standard curve.

Myeloperoxidase (MPO) activity was assessed, as reported by Desser *et al*. [40]. In the presence of hydrogen peroxide, MPO catalyzes the oxidation of guaiacol to oxidized guaiacol. Guaiacol in its oxidized form has a brown color which is measured photometrically at 470 nm wavelength. The generated color intensity is proportional to the amount of oxidized guaiacol produced in the reaction.

Superoxide dismutase (SOD) activity was determined as previously documented [41,42]. 50 µL of the mitochondrial fraction was diluted in distilled water to make a 1 in 10 dilutions. An aliquot of 0.2 mL of the diluted sample was added to 2.5 mL of 0.05M carbonate buffer (pH 10.2) to equilibrate in the spectrophotometer and the reaction started by the addition of 0.3 mL of freshly prepared 0.3 mM adrenaline to the mixture which was quickly mixed by inversion. The reference cuvette contained 2.5 mL buffer, 0.3 mL of the substrate (adrenaline) and 0.2 mL of water. The increase in absorbance at 480 nm was monitored every 30 s for 150 s.

Glutathione peroxidase (GPx) activity was determined as documented by Rotruck *et al*. [43] with some modifications. Briefly, the reaction mixture containing the epididymal sperm suspension was incubated at 37°C for 3 min after which 0.5 mL of 10% trichloroacetic acid (TCA) was added and then centrifuged at 3000 rpm for 5 min. 2mL of phosphate buffer and 1mL of 5'-5'- dithiobis-2-dinitrobenzoic acid (DTNB) solution was added to 1mL of each of the supernatants. Absorbance was read against a blank at 412 nm. GPx activity was observed by plotting the standard curve.

### Assessment of sperm DNA fragmentation, oxidative DNA damage and apoptosis

Assay of sperm DNA fragmentation was carried out as previously described [44,45] using aniline blue staining method. Briefly, 10 μl of washed spermatozoa was spread onto the slides. Dried smears were fixed in 3% buffered glutaraldehyde in 0.2 M phosphate buffer (pH 7.2) for 30 min. Slides were stained with1% aniline blue mixed with 4% acetic acid (pH 3.5) for 5 min. Then the staining slides were washed in running water for at least 3 min and de­hydrated in a graded ethanol for 2 min in each step. Finally, slides were cleared by xylene for at least 30 min and were mounting by a drop of Entelan and were dried overnight at room temperature. At least 200 sperm cells per slide were evaluated under light micros­copy with an objective lens (×100) and the percentage of stained sperm heads was calculated.

8-hydroxy-2′-deoxyguanosine (8OHdG), an index of oxidative sperm DNA damage, was determined using standard ELISA kit (Elabscience Biotechnology Co., Ltd, USA; product numbers: E-EL-0028) following the manufacturer’s manual. The standard working solution was added to the first two columns: each concentration of the solution was added in duplicate, to one well each, side by side (50 uL for each well). The mitochondrial fraction was added to the other wells (50 uL for each well). Immediately, 50 μL of Biotinylated Detection Ab working solution was added to each well. The plate was after that covered with a sealer and incubated at 37°C for 45 min. It was ensured that the solutions were added to the bottom of the micro ELISA plate well. Also, contact with the inside wall was avoided. The solution was thereafter aspirated from each well, and 350 uL of wash buffer added to each well. These were soaked for 1–2 min, and the solution was aspirated from each well, then patted dry against clean absorbent paper. The wash step was repeated three times. 100 μL of HRP Conjugate working solution was added to each well and covered with the plate sealer then incubated for 30 min at 37°C. The solution from each well was aspirated, and the wash process was repeated five times. Then 90 μL of Substrate Reagent was added to each well and covered with a new plate sealer then incubated for another 15 min at 37°C. The plate was protected from light. The 50 μL of stop solution was added to each well. The optical density value of each well was determined with a microplate reader set to 450 nm.

The activity of Caspase 3, a marker of apoptosis, was determined as described for 8OHdG using standard ELISA kit (Elabscience Biotechnology Co., Ltd, USA; product number: E-EL-RB0656, respectively) following the manufacturer’s manual.

### Estimation of serum and testicular levels of testosterone

Serum and testicular testosterone were determined using the ELISA kit (Monobind Inc. USA; product number: 4806-300A) following the manufacturer’s manual as previously reported [46]. Briefly, for serum testosterone assay, 10 µL of standards, control and serum samples were dispensed into their respective wells. 100 µL Testosterone-HRP conjugate was added to each well. Substrate blank was dispensed into well A1. The wells were then covered with foil. Then incubation was carried out for 1 h at room temperature, the foil was removed and well contents aspirated. Then 300 µL diluted wash solution was used three times to wash the wells. The soak time between each wash cycle was more than 5 s. The remaining fluid was carefully removed by tapping the strips on tissue paper. 100 µL of TMB substrate solution was added into all wells. The wells were incubated for 15 min at room temperature in the dark. 100 µL stop solution was dispensed into all wells in the same order and the same rate as for the substrate. The absorbance of the specimen was read at 450 nm within 20 min after addition of the stop solution.

Testicular testosterone measurement was determined similarly; however, the testes supernatant obtained from testicular homogenate was used instead of the serum.

### Statistical analysis

Statistical Package for Social Sciences (SPSS, version 16) was used to analyse the data. One-way analysis of variance (ANOVA) followed by Tukey’s post hoc test for pairwise comparison was used to evaluate the differences between variables. Pearson’s bivariate correlation followed by multivariate regression analysis was done to assess the correlation between sperm quality, sperm DFI, 8-OHdG, and caspase 3. Data are expressed as mean ± SD. Values with *p* < 0.05 were adjudged significantly different.

## Results

### Sperm variables

Tables 1 and 2 display the results of classic semen analysis. Compared with the control, codeine-treated animals had significantly more reduced sperm concentration, reduced motility and viability (*p* < 0.05). Codeine treatment also led to more inferior sperm morphology with various sperm abnormalities (*p* < 0.05). These changes were observed to be dose-dependent.

**Table 1.** Effect of chronic codeine use on sperm parameters.

Variables	Control	Low-dose codeine	High-dose codeine
Sperm concentration (×106/ml)	67.14 ± 1.57^a^	48.43 ± 4.42^b^	43.00 ± 3.69^c^
Sperm motility (%)	91.71 ± 2.36^a^	67.57 ± 4.64^b^	55.71 ± 5.99^c^
Sperm viability (%)	85.43 ± 5.47^a^	66.87 ± 6.09^b^	52.71 ± 2.98^c^

Same parameter carrying different alphabets (a,b,c) are statistically different at *P* < 0.05.

**Table 2.** Effect of chronic codeine use on sperm morphology.

Variables	Control	Low-dose codeine	High-dose codeine
Tailless head	3.86 ± 0.69^a^	7.57 ± 1.39^b^	10.43 ± 1.13^c^
Headless tail	3.57 ± 0.97^a^	7.43 ± 1.39^b^	10.85 ± 0.89^c^
Rudimentary tail	1.71 ± 0.95^a^	7.28 ± 1.11^b^	10.71 ± 1.38^c^
Bent tail	5.86 ± 1.67^a^	14.42 ± 0.97^b^	15.00 ± 1.00^b^
Curved tail	5.14 ± 1.21^a^	12.28 ± 1.25^b^	14.71 ± 0.95^c^
Looped tail	2.00 ± 0.81^a^	8.00 ± 0.81^b^	10.71 ± 0.75^c^
Bent midpiece	6.28 ± 1.49^a^	13.00 ± 1.73^b^	14.14 ± 1.21^b^
Curved midpiece	7.71 ± 0.95^a^	13.71 ± 1.70^b^	15.28 ± 0.75^c^
Percentage of abnormal sperm (%)	8.95 ± 1.02^a^	23.46 ± 1.92^b^	31.25 ± 2.05^c^
Percentage of normal sperm (%)	91.02 ± 1.01^a^	76.53 ± 1.92^b^	68.75 ± 2.05^c^

Same parameter carrying different alphabets (a,b,c) are statistically different at *P* < 0.05.

### Sperm lipid peroxidation and anti-oxidants

The effect of codeine on oxidative balance in sperm cells is shown in Table 3. Codeine treatment led to significantly higher concentration and activity of MDA and MPO respectively in sperm cells (*p* < 0.05). Dose-dependent significantly lower activities of SOD and GPx were also observed in codeine-treated rabbits *(p* < 0.05).

**Table 3.** Effect of chronic codeine use on sperm oxidative markers and anti-oxidants.

Variables	Control	Low-dose codeine	High-dose codeine
MDA (nmol/ml)	5.04 ± 0.14^a^	7.08 ± −0.13^b^	8.15 ± 0.17^c^
MPO (U/mg)	54.52 ± 1.34^a^	77.12 ± 2.39^b^	86.25 ± 1.79^c^
SOD (U/mg)	825.43 ± 4.31^a^	786.43 ± 2.43^b^	740.29 ± 3.40^c^
GPx (U/mg)	420.00 ± 3.10^a^	369.86 ± 6.86^b^	320.10 ± 4.34^c^

Same parameter carrying different alphabets (a,b,c) are statistically different at *P* < 0.05.

### Serum and testicular testosterone

**Figure 1.**
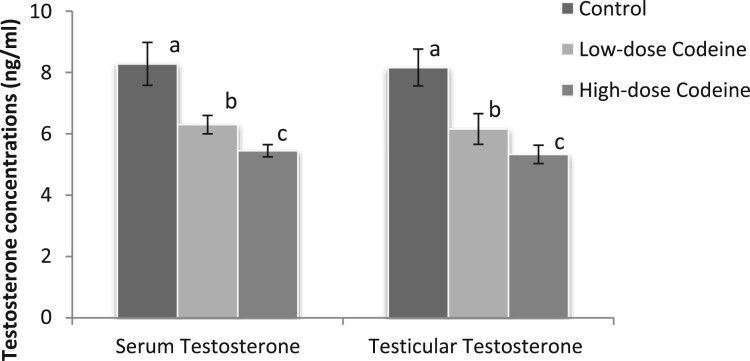
Effect of chronic codeine use on serum and testicular testosterone. Same parameter carrying different alphabets (a,b,c) are statistically different at *P* < 0.05.

The effect of codeine on serum and testicular levels of testosterone is shown in Figure 1. Codeine treatment led to significant suppression of circulating and testicular levels of testosterone (*p* < 0.05). The decline in the androgen level was dose-dependent *(p* < 0.05).

### Sperm plasma membrane integrity

**Figure 2.**
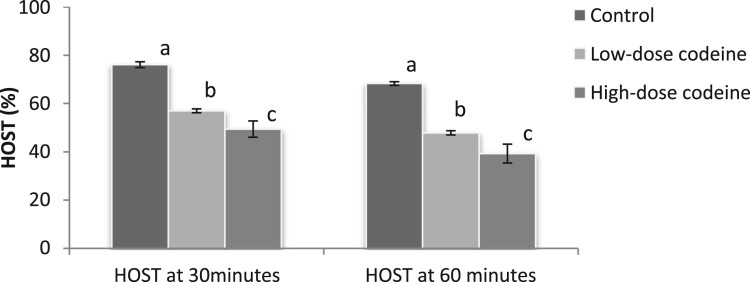
Effect of chronic codeine use on sperm membrane integrity using HOST. Same parameter carrying different alphabets (a,b,c) are statistically different at *P* < 0.05.

Figure 2 shows the effect of codeine treatment on sperm plasma membrane integrity using the hypo-osmotic swelling test (HOST). At 4 mg/kg b.w, codeine led to 25% and 30% decline in sperm plasma membrane integrity in 30 and 60 min respectively. At 10 mg/kg b.w, this molecule caused 35% and 42% decline in sperm plasma membrane integrity in 30 and 60 min respectively. The poor membrane integrity observed in codeine treatments were dose-dependent and significant when compared with the control (*p* < 0.05).

### Sperm DNA fragmentation, oxidative DNA damage and apoptosis

**Figure 3.**
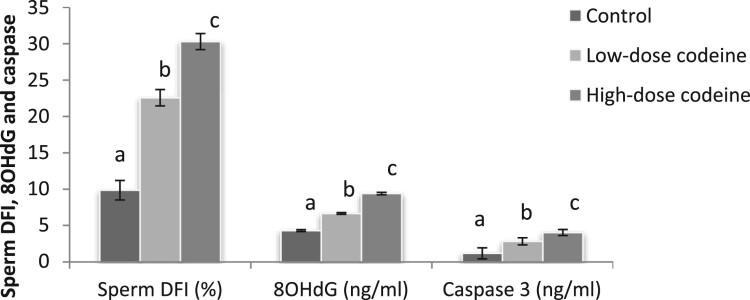
Effect of chronic codeine use on sperm DFI, 80HdG, and caspase3. Same parameter carrying different alphabets (a,b,c) are statistically different at *P* < 0.05.

Sperm DNA fragmentation index (DFI) was significantly higher in codeine-treated animals (Figure 3). Low-dose codeine treatment led to a 56% rise in sperm DFI while high-dose codeine led to a 67% rise.

Similarly, significantly higher levels of 8-hydroxy-2′-deoxyguanosine and caspase 3 activity were seen in codeine-treated groups when compared with the control (Figure 3). Low-dose and high-dose codeine led to 35% and 54% higher levels of 8OHdG respectively, and 58% and 71% higher levels of caspase 3 activities respectively.

### Relationship between sperm quality, sperm DNA fragmentation, 8OHdG and caspase 3 activity

Comparisons of sperm quality, 8OHdG and caspase 3 activity between the control and codeine-treated rabbits are shown in Table 4. 8OHdG was negatively correlated with sperm concentration (*r* = −0.904; *p* < 0.001), sperm motility (*r* = −0.934; *p* < 0.001), sperm viability (*r* = −0.935; *p* < 0.001), percentage of normal sperm (*r* = −0.961; *p* < 0.001), HOST at 30 min (*r* = −0.938; *p* < 0.001) and HOST at 60 min (*r* = −0.942; *p* < 0.001), and positively correlated with sperm DNA fragmentation (*r* = 0.974; *p* < 0.001). Similarly, caspase 3 activity was negatively correlated with sperm concentration (*r* = −0.836; *p* < 0.001), sperm motility (*r* = −0.853; *p* < 0.001), sperm viability (*r* = −0.857; *p* < 0.001), percentage of normal sperm (*r* = −0.872; *p* < 0.001), HOST at 30 min (*r* = −0.879; *p* < 0.001) and HOST at 60 min (*r* = −0.894; *p* < 0.001), and positively correlated with sperm DNA fragmentation (*r* = 0.903; *p* < 0.001). Although, these correlations were significant (*p* = 0.000), they were higher between O8HdG and sperm quality than between caspase 3 activity and sperm quality.

**Table 4.** Correlations observed between 8OHdG, caspase and sperm quality.

Variables	8OHdG	Caspase 3 activity	*P* value
Sperm concentration	−0.904	−0.836	0.000
Sperm motility	−0.934	−0.853	0.000
Sperm viability	−0.935	−0.857	0.000
Percentage of normal sperm	−0.961	−0.872	0.000
HOST at 30 min	−0.938	−0.879	0.000
HOST at 60	−0.942	−0.894	0.000
Sperm DNA Fragmentation	0.974	0.903	0.000

Multivariable regression analysis revealed that oxidative sperm DNA damage, evaluated by sperm 8OHdG, was more associated with codeine-induced sperm DNA fragmentation (z-score = 0.835; *p* = 0.000) than sperm apoptosis (z-score = 0.156; *p* = 0.187) (Table 5).

**Table 5.** Multivariate regression analysis to assess the association between oxidative DNA damage and apoptosis, and sperm DNA fragmentation.

	B	SE	Βeta	T	*p*
Sperm 8OHdG	3.405	0.464	0.835	7.343	0.000
Sperm caspase 3 activity	1.023	0.747	0.156	1.370	0.187

B: unstandardized regression coefficient; SE: standard error for the unstandardized regression coefficient; Beta (*β*): standardized regression coefficient; *t*: t test statistic; *p*: probability value.

## Discussion

**Figure 4.**
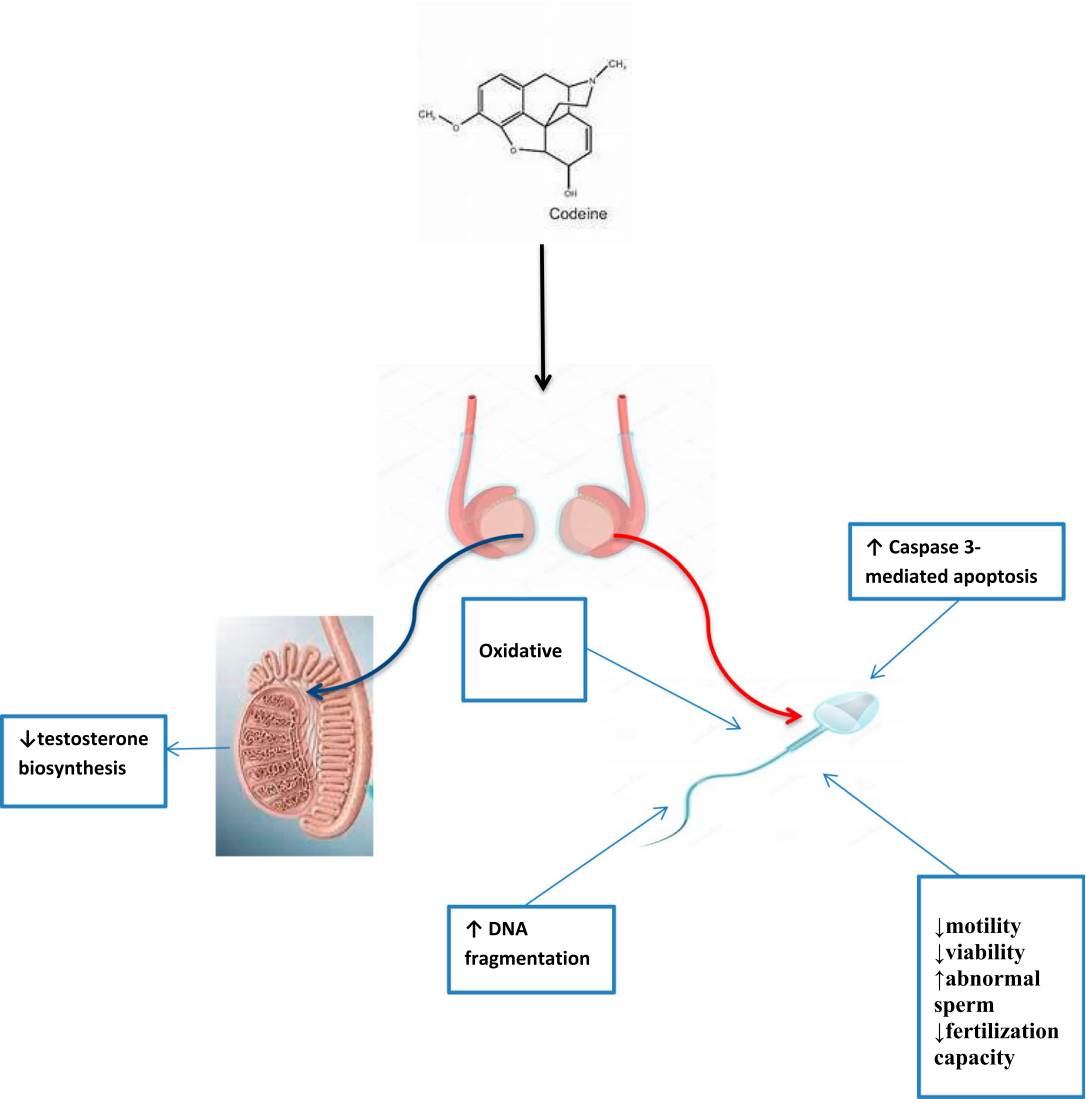
Proposed mechanism of the effect of codeine on sperm cell.

Despite the increases in incidences of male factor infertility of unknown causes and codeine abuses, data reporting the impact of codeine on male reproduction remains scarce. Although in our previous studies, we demonstrated that oral codeine exposure in rabbits impaired fertility indices [30], and led to testicular degeneration and testosterone suppression [46]. This present study is an extension of our previous studies on [30,46] and data sets were obtained separately. This study demonstrated for the first time that chronic codeine use is strongly associated with poor sperm quality and sperm DNA fragmentation (Figure 4). We have also demonstrated that codeine-induced DNA fragmentation observed in the study is more associated with oxidative DNA damage than caspase-mediated apoptosis.

Semen analysis is a series of tests that assess the functions of male reproductive organs/glands and systems [47] and is an indicator of sperm quality. Sperm concentration assesses testicular function while sperm morphology evaluates the toxic effect of exposures to sperm cells [48,49]. The present study revealed that chronic use of codeine led to poor sperm quality evident by reduced sperm concentration, motility, viability and percentage of sperm with normal morphology. These findings align with previous studies [50–52] that reported that morphine use led to significantly reduced sperm viability, morphology, count and motility. Our observation that codeine led to poor sperm quality is also in agreement with a previous study [25] that associated the use of tramadol with poor sperm quality. The reduced sperm count observed following codeine use is due to, at least in part, the reduced circulating and testicular concentrations of testosterone observed following the use of the molecule via suppression of spermatogenesis [53]. It has been established that optimal levels of serum and testicular testosterone are essential for spermatogenesis; hence, codeine-induced testosterone suppression possibly led to impaired spermatogenesis and consequently reduced sperm count. Our findings may also corroborate the reports of the National Toxicology Program (NTP) [54]. Following 14 days of oral codeine exposure, testicular degeneration was observed in rats. Although, it was not stated whether or not sperm parameters were evaluated on day 14, the testicular degeneration observed could account for the codeine-induced poor sperm quality observed in our study. Strikingly, after 13 weeks of codeine exposure, no significant differences were observed in sperm morphology between control and exposed rats. This might be due to an adaptation mechanism with possible testicular regeneration and normal spermatogenesis.

The hypo-osmotic swelling test (HOST) assesses the membrane integrity of the sperm cells. It identifies individual sperm cell with minimal DNA fragmentation [55], assesses sperm functional ability [36,56], and it is a pointer to the fertility capacity of the spermatozoa [57,58]. Our finding that codeine led to a dose-dependent decrease in sperm membrane integrity suggests that chronic codeine use impairs the fertility capacity of sperm cells.

The plasma membrane of the sperm cell is rich in polyunsaturated fatty acids (PUFA) which predisposes it to oxidation by reactive oxygen species (ROS) [59–61]. ROS causes lipid peroxidation of the sperm membrane, thus promoting alteration in membrane fluidity leading to decline in sperm motility [62,63]. It also attacks the mitochondrial and nuclear DNA leading to increased sperm DNA fragmentation [64,65], and apoptosis [66,67]. This present study revealed that chronic codeine use caused significant increase in sperm MDA and MPO activity and significant reduction in sperm SOD and GPx activities leading to oxidative damage of the sperm cells. There was also an increase in oxidative sperm DNA damage, evident by a rise in 8OHdG. This was associated with significant increase in caspase 3-mediated apoptosis and sperm DNA fragmentation. The codeine-induced sperm oxidative damage observed in this study is in tandem with previous studies [25,49,52] that reported a rise in lipid peroxidation index and decline in antioxidant following opioid use. This lipid peroxidation might account for the decrease in sperm plasma membrane integrity as evaluated by HOST. Oxidation results in damaged ionic transport systems which lead to difficulty in maintaining the integrity of ionic gradient [58]. The codeine-induced lipid peroxidation also explains the reduction in sperm motility [68], viability and morphology observed in this study.

Sperm DNA integrity is essential for fertilization, embryo development, and transmission of genetic material to the offspring [69]. DNA fragmentation is the most frequent DNA anomaly associated with poor sperm quality, low fertilization rates, and impaired embryo quality [69]. Sperm DNA fragmentation is usually caused either by oxidative DNA damage and apoptosis [69–71]. Our observation in this study suggests that codeine-induced sperm DNA fragmentation is due to both oxidative damage and caspase 3-dependent apoptosis.

A strong negative correlation between sperm quality, and oxidative sperm damage and caspase 3-mediated apoptosis was observed. Also, a positive correlation was observed between sperm DNA fragmentation, oxidative sperm damage and caspase 3-mediated apoptosis. This correlation revealed that the poor sperm quality and increased sperm DNA fragmentation observed following codeine administration is due to both oxidative damage and apoptosis. It is worthy to note that the correlation was much stronger between sperm DNA fragmentation and oxidative DNA damage than sperm DNA fragmentation and caspase 3 activity. In addition, multivariable regression studies revealed that oxidative DNA damage rather than caspase 3 activity is a determinant of codeine-induced sperm DNA fragmentation. These possibly infer that codeine-induced sperm DNA damage predominantly by oxidative damage.

## Conclusion

Summarily, the results of the present study show that chronic oral exposure to codeine causes sperm DNA fragmentation and poor sperm quality via oxidative damage and caspase-mediated apoptosis, which is associated with impaired sperm fertilization capacity. Findings of the present study may be significant in explaining drug-induced male factor infertility, particularly, those associated with opioid use.

## Declarations

### Ethics approval and consent to participate

Ethical approval was obtained from the Ethics Review Committee, Ministry Health, Oyo State, Nigeria, with Reference NumberAD13/479/1396. The experiment followed the US NAS guidelines.
